# *Aedes albopictus* Mosquito: The Main Vector of the 2007 Chikungunya Outbreak in Gabon

**DOI:** 10.1371/journal.pone.0004691

**Published:** 2009-03-04

**Authors:** Frédéric Pagès, Christophe N. Peyrefitte, Médard Toung Mve, Fanny Jarjaval, Sylvain Brisse, Isabelle Iteman, Patrick Gravier, Dieudonné Nkoghe, Marc Grandadam

**Affiliations:** 1 Institut de médecine tropicale du Service de santé des armées, Unité d'entomologie médicale, URMITE UMR 6236, Marseille, France; 2 Institut de médecine tropicale du Service de santé des armées, Unité de virologie tropicale, Marseille, France; 3 Ministère de la Santé publique, Libreville, Gabon; 4 Institut Pasteur, plate-forme génomique, Paris, France; 5 Service médical du 6e BIMA, Libreville, Gabon; University of Sydney, Australia

## Abstract

The primary vector at the origin of the 2007 outbreak in Libreville, Gabon is identified as *Aedes albopictus*, trapped around the nearby French military camp. The Chikungunya virus was isolated from mosquitoes and found to be identical to the A226V circulating human strain. This is the first field study showing the role of the recently arrived species *Aedes albopictus* in Chikungunya virus transmission in Central Africa, and it demonstrates this species' role in modifying the epidemiological presentation of Chikungunya in Gabon.

## Introduction

First isolated in Tanzania in 1952, Chikungunya virus (CHIKV), an *Alphavirus* member of the *Togaviridae*, is now a world-wide public health problem [1], [2]. A CHIKV outbreak began in Kenya in 2004, spread to populated islands in the Indian Ocean and later jumped to India, and Europe [3]–[6]. Though previously considered to be a vector with poor efficiency, *Ae. albopictus* was identified as the major vector in La Reunion Island and Europe [2], [7]. Sequence analysis of the virus genome revealed that these recent outbreaks were caused by a new variant characterized by a mutation in the E1 envelope glycoprotein gene (A226V) [4]. This mutation has favored improved transmissibility of the virus by the mosquito *Ae. albopictus* [8].

In Africa, while recent serological surveys suggest a high prevalence of *Togaviridae*, *Flaviviridae* and *Bunyaviridae* [9] and there is recent evidence of CHIKV circulation in Republic Democratic of Congo [10] and Cameroon [11], detailed information about CHIKV circulation remains imprecise. The Chikungunya virus was classically isolated from several sylvatic *Aedes sp.* including *Aedes africanus*, *Aedes furcifer*, *Aedes luteocephalus*, *Aedes neoafricanus*, *Aedes taylori* [12], [13]. Rural outbreaks were found to be heavily dependent upon the densities of these sylvatic mosquitos, which increase during periods of heavy rainfall. During the late 1970s, serological surveys conducted in Gabon revealed a CHIKV exposure seroprevalence of 8.5% in urban Libreville (the capital of Gabon), rising from 20% to 44% in rural areas [14]. In recent times, the Asian tiger mosquito *Aedes albopictus* has succeeded in colonizing some parts of sub-Saharan Africa. In central Africa, this spreading vector was first collected in 2000 in Cameroon, in 2003 in Equatorial Guinea, and in 2006 in Gabon [15]. During the last 6 years, the urban cycle has tended to play an increasing role in CHIKV transmission in Central Africa [11], bringing into question the participation of urban *Aedes sp.* such as *Aedes aegypti* or the recently discovered *Aedes albopictus* as a primary vector.

**Figure 1:**
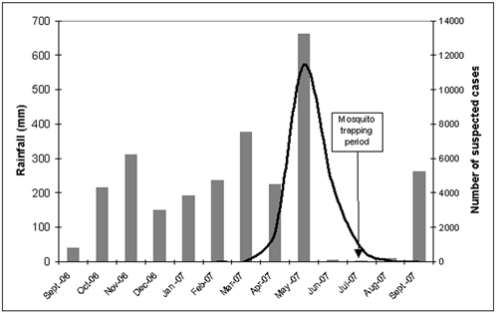
Rainfall in Libreville, Gabon from September 2006 to September 2007 (grey bars) and monthly numbers of suspected Chikungunya cases in Gabon, March 2006–September 2007 (black curve).

In April 2007, an outbreak of Dengue-like symptoms occurred in Libreville where *Aedes albopictus* was first detected in December 2006. The majority of patients were presumptively treated for malaria with no symptom improvement. When malaria diagnosis tests (blood thick smear, slide or malaria Core® rapid test) were performed, the results were negative and CHIKV was identified from patient blood samples as the main causative agent [16]. The Gabon Ministry of Public Health commissioned a crisis committee to manage the outbreak; patients were identified as suspected CHIK cases if they presented with a sudden fever over 39°C which lasted for more than 48 h in spite of anti-malarial treatment, in addition to a headache and severe joint and muscle pain, with or without a rash. From May 2007 through September 2007, 13,802 suspected cases were recorded in health centers and 829 patients were hospitalized, though fortunately no deaths occurred. From April 2007 through May 2007, 5,000 additional cases were retrospectively included by the Gabon health authorities after further analysis (Figure 1). The transmission decrease observed in June was presumably due to the end of the rains and corresponding decrease in vector density—both *Aedes albopictus* adult abundance and oviposition rate are closely correlated with rainfall [17]. During the peak of the May outbreak, CHIKV transmission was also recorded in Bitam, the capital of the Woleum Ntem province (north Gabon). Since August 2007, no suspected cases have been recorded in Libreville despite the start of the rainy season in September of that year. However, new CHIK cases were confirmed in November 2007 in Lastourville, Ogoue Lolo province (south-east Gabon).

In Libreville, *Aedes albopictus* and *Aedes aegypti* are potential vectors of CHIK and DEN viruses. To assess the respective roles of *Aedes albopictus* and *Aedes aegypti* in Dengue and chikungunya transmission in Libreville, we proceeded in two steps. An experimental study was conducted in the laboratory, using experimental infections to determine the ability of *Ae. albopictus* and *Ae. aegypti* collected in Libreville to transmit CHIK and Dengue virus [18]. *Ae. albopictus* showed a higher susceptibility to CHIK virus than *Ae. aegypti*. Thus, *Ae. albopictus* appears to be a better vector for CHIK virus than *Ae. aegypti*. Moreover, as demonstrated by the rates of disseminated infection obtained, Gabonese *Ae. albopictus* was less susceptible to DEN virus, leading us to consider *Ae. albopictus* as a secondary dengue vector. During the same period, a field study was conducted to research DENV- and CHIKV-infected mosquitoes. The objective of the present study is to identify the primary vector at the origin of the Libreville outbreak.

## Methods

**Figure 2:**
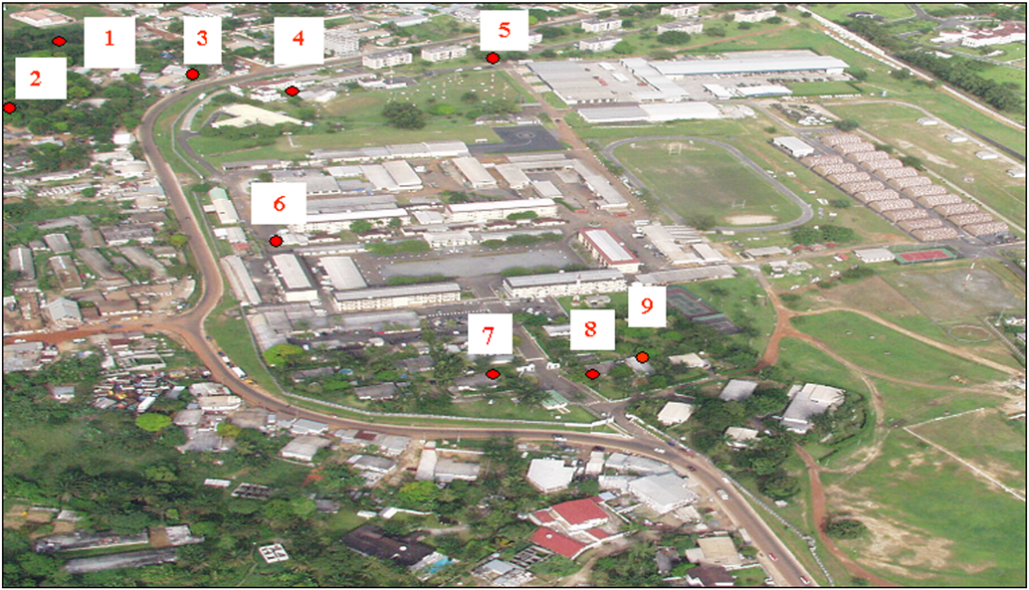
Location of mosquito trapping sites both inside [4]–[9] and outside [1]–[3] the military camp of French armed forces in Libreville, Gabon.

Bg-sentinel odor traps (Biogents AG Regensburg, Germany) were used in the French military camp of Libreville (lat: 0.44 long: 9.43) and its neighboring areas (Figure 2). Traps were set up during the entire day for 15 days. Catch bags were collected each morning (7 am) and evening (8 pm). Mosquito identifications were carried out using one of several keys [19], [20], [21]. The average numbers of *Aedes sp.* caught inside and outside the camp were compared using Student's *t*-test. Each day's collections of *Aedes* mosquitoes were sorted by trap, species and sex. Mosquito pools were mixed with sterile PBS, crushed and 0.22 µm filtered (disposable 0.22 µm filter, Dutscher Dominique, 67172 Brumath, France). For samples of fewer than 5 mosquitoes, a PBS volume of 200 µl was used for crushing; for larger samples, the volume was increased to prevent the mixture becoming too viscous. Genomic CHIKV detection was performed for each pool using TaqMan RT-PCR [22], while Dengue virus genome detection was conducted using a previously described method [23]. To isolate the virus, the supernatants of each genomic CHIKV positive pool were inoculated onto Vero E6 and C6/36 cell lines [22]. The CHIKV genomes were partially sequenced (E1-30UTR junction) and compared to human isolates collected during this outbreak (GenBank accession numbers EF613342, EF613343, EF613344); alignment was performed with ClustalW1.7 software.

## Results

During the two weeks studied in July 2007, a total of 10,605 mosquitoes were caught: *Culex quinquefasciatus* (5642 females and 3807 males), *Mansonia uniformis* (78 females and 4 males), *Anopheles gambiae* (7 females and 1 male), *Anopheles sp.* (8 females and 1 male), *Aedes sp.* (10 females), 1046 *Aedes albopictus* (801 females and 245 males) and 80 *Aedes aegypti* (71 females and 9 males). The average number of *Ae. albopictus* specimens per trap caught inside and outside the camp were 1.7 and 16.3, respectively (*p*<0.0001). There was no statistical difference between the average number of *Ae. Aegypti* specimens caught inside and outside the camp. Pool sizes and compositions are given in Table 1, with an average pool size of 3.81, and a range from 1 to 41. Neither the *Aedes aegypti* nor *Aedes albopictus* pools were positive for Dengue virus. The two Aedes *albopictus* female pools (N = 21, N = 12) were positive for CHIKV according to genome detection by TaqMan RT-PCR, and Chikungunya virus was also isolated when their filtered supernatants were inoculated onto Vero E6 and C6/36 cell lines. The female *Aedes albopictus* minimal infection rate (calculated as the ratio of positive pools to the total number of *Aedes albopictus* female mosquitoes tested) was approximately 2.5‰. The CHIKV genomes were partially sequenced (GenBank accession number EU403052). When compared to the human isolates from blood samples of Gabonese patients [16], the *Aedes albopictus* isolated CHIKV displayed 98.7% to 99.1% identity at the nucleotide level and 99.4% to 99.6% identity at the amino-acid level. Both human and mosquito isolates displayed the A226V mutation within the E1 gene [4].

**Table 1:** Total number of *Aedes albopictus* and *Aedes aegypti* trapped by location both inside [4]–[9] and outside [1]–[3] the French military camp, Libreville, Gabon, July 2007.

trap number	*Aedes aegypti*		*Aedes albopictus*	
	females	males	females	males
**1**	9	0	373^a^	48
**2**	12	2	257^b^	149
**3**	17	4	38	30
4	5	0	6	2
5	4	0	7	0
6	7	0	9	1
7	5	2	25	1
8	6	1	48	10
9	6	0	38	4
All traps	71	9	801	245

a one pool (21 mosquitoes) infected by CHIKV (July 14^th^ 2007).

b one pool (12 mosquitoes) infected by CHIKV (July 20^th^ 2007).

## Discussion

Sequence analysis of the virus genome revealed that the recent CHIKV outbreaks were caused by a new variant characterized by a mutation in the E1 envelope glycoprotein gene (A226V) [4]. The A226V mutation has favored a better transmissibility of the virus by the mosquito *Ae. albopictus* [8]. In Gabon, we found the same virus vector association as detected during the 2006 Réunion Island Outbreak: A226V mutated CHIKV and an *Aedes albopictus* strain. Interestingly, an analysis of full-length viral sequences reveals three independent instances of *Ae. albopictus* exposure to the virus in India, Cameroon and Gabon, each followed by the acquisition of a single adaptive mutation providing a selective advantage for transmission by this mosquito [24]. Therefore, the A226V mutation is strongly believed to play a role in the epidemiological success of CHIKV in Gabon.

Inside the camp, the vector control program against *Aedes* mosquitoes is based on the destruction or elimination of unwanted natural and artificial water containers. The efficacy of this strategy is highlighted by the lower average number of *Ae. albopictus* caught in traps inside the camp. This study confirms that reducing the number of such containers in and around homes significantly decreases the size of *Ae. albopictus* populations.

The low number of *Aedes aegypti* captured did not allow us to determine if this species had participated as a second vector for CHIKV transmission during the outbreak. In Libreville, *Ae. aegypti* and *Ae. albopictus* are often sampled in the same container [15], but *Ae. albopictus* exploits a wider range of breeding sites than *Ae. aegypti*, which is classically more dependent on artificial breeding sites than *Ae. albopictus*. During the dry season, the reduction of artificial breeding sites has a stronger impact on *Ae. aegypti* population size. Our field study is not able to clarify the role of *Ae. aegypti* in CHIKV transmission during the outbreak nor to identify *Ae. aegypti* as the DEN vector in Libreville. Nevertheless, the results of experimental infection suggest that *Ae. aegypti* was probably a better DEN vector in Libreville [18]. *Ae. albopictus* was a more efficient experimental vector of CHIK virus than *Ae. aegypti. formosus*, and is assumed to be a better CHIK vector in the field, not playing a significant role in DEN transmission in Gabon.

The high level of identity between the human and mosquito virus isolates from Libreville indicates a strong relationship between these CHIKV isolates, while the finding of CHIKV infected *Aedes albopictus* during the middle of the dry season and the high experimental infection rate highlight the role of this mosquito as the primary vector during the 2007 outbreak in Gabon. This was the first field study showing the role of recently arrived *Aedes albopictus* in the transmission of Chikungunya virus in Central Africa. *Aedes albopictus* has currently been collected in four of the Gabon's nine provinces, indicating an ongoing colonization of the country similar to the colonization of Cameroon [25]. Interestingly, epidemic CHIKV transmission only occurred in the major population centers of these provinces, such as Libreville (Estuaire Province), Bitam (Woleum Ntem Province) and Lastourville (Ogoue Lolo Province), emphasizing the role of *Aedes albopictus* in the modification of Chikungunya epidemiological presentation in Gabon from rural and endemic to urban and endemo-epidemic. This trend was observed as early as 2000 in outbreaks in the Democratic Republic of the Congo [10]. As *Ae. albopictus* continues to spread, displacing *Ae. aegypti* in African countries like Cameroon [25], the occurrence of this vector in the African rainforest is of concern, as yellow fever and other arboviruses are endemic in central Africa. In Gabon, *Ae. albopictus* should not be an efficient DEN vector [18], but we cannot forecast the results as it encounters West Nile virus, Sindbi virus, Orungo virus or Semliki forest virus [26], [27].
