# Correction: *Aedes albopictus* Mosquito: The Main Vector of the 2007 Chikungunya Outbreak in Gabon

**DOI:** 10.1371/annotation/4145c2b9-dca1-4eef-996a-8e79de4dc1f5

**Published:** 2009-05-27

**Authors:** Frédéric Pagès, Christophe N. Peyrefitte, Médard Toung Mve, Fanny Jarjaval, Sylvain Brisse, Isabelle Iteman, Patrick Gravier, Hugues Tolou, Dieudonné Nkoghe, Marc Grandadam

Dr. Hugues Tolou should be included in the byline as the eighth author. He is affiliated with the Unité de Virologie Tropicale, Institut de Médecine Tropicale du Service de Santé des Armées, Marseille, France. His contributions were as follows: Contributed reagents/materials/analysis tools.

